# A Series of Personalized Melatonin Supplement Interventions for Poor Sleep: Feasibility Randomized Crossover Trial for Personalized N-of-1 Treatment

**DOI:** 10.2196/58192

**Published:** 2025-09-26

**Authors:** Mark J Butler, Thevaa Chandereng, Heejoon Ahn, Stefani Slotnick, Danielle Miller, Alexandra Perrin, Jordyn Rodillas, Ciaran P Friel, Ashley M Goodwin, Ying Kuen Cheung, Karina W Davidson

**Affiliations:** 1 Northwell, 2000 Marcus Ave, Suite 300, New Hyde Park, NY, 11042-1069, United States, 1 6467667181; 2 Institute of Health System Science, Feinstein Institutes For Medical Research, Manhasset, NY, United States; 3 Mailman School of Public Health, Columbia University, New York, NY, United States

**Keywords:** personalized trial, personalized, N-of-1, sleep quality, poor sleep, sleep duration, virtual, melatonin, placebo, feasibility, intervention, randomized crossover trial, heterogeneity, effectiveness, sleep health, Fitbit, wearable

## Abstract

**Background:**

Poor sleep (defined by short sleep duration or poor quality) is a common condition with potential serious health consequences. Exogenous melatonin supplements have been found to effectively improve poor sleep but have also been shown to have heterogeneity of treatment effects (HTEs) between individuals. Personalized N-of-1 trials, in which each participant is the unit of analysis, are ideal for identifying whether a treatment with high HTE is beneficial for each individual patient.

**Objective:**

This study aimed to identify the feasibility, acceptability, and effectiveness of a series of personalized N-of-1 trials of melatonin for poor sleep.

**Methods:**

This study consisted of 60 digital, personalized N-of-1 crossover trials comparing the effects of 3.0 mg and 0.5 mg of melatonin versus placebo for poor sleep with randomization to 1 of 2 orders. The trial comprised a 2-week baseline period and a 12-week intervention period. The primary outcomes were usability of the personalized trial system (measured using the System Usability Scale [SUS]) and participant satisfaction with the trial. Effectiveness outcomes included sleep duration (measured using a Fitbit activity tracker [Google]) and sleep quality (measured using the consensus sleep diary).

**Results:**

Participants rated the usability of the personalized trial as acceptable (average SUS score 76.3, SD 17.1), and 96% (55/57) of those who completed satisfaction surveys stated that they would recommend the trial to others. Importantly, indices of HTE were low for 3.0 mg and 0.5 mg doses of melatonin, indicating that the effect of these treatments on sleep duration and sleep quality did not substantially vary between participants and that averaged treatment responses are appropriate. Averaged participant sleep duration did not significantly differ between the 3.0 mg (*P*=.70) and 0.5 mg (*P*=.90) melatonin intervention periods and the baseline period. In addition, regression models did not show differences between different levels of melatonin and placebo periods for sleep duration or quality.

**Conclusions:**

Participant ratings of the usability of and satisfaction with this series of personalized N-of-1 trials of melatonin for sleep suggest these trials are both feasible and acceptable. However, our results show that melatonin supplements did not significantly improve sleep duration or sleep quality. Furthermore, the treatment effects’ lack of heterogeneity among participants suggests that future use of N-of-1 trials of melatonin for poor sleep is not needed.

## Introduction

Sleep health is a multidimensional construct that can be difficult to define. Healthy sleep can be defined in terms of sleep duration, sleep quality, and the subjective experience of restful sleep, among other aspects [1]. Although most sleep research focuses on discrete sleep disorders [1,2], difficulties with sleep are extremely common [3,4]. Recent research has shown that short sleep duration (≤6 per night) is prevalent within 37.6% of US workers while poor sleep quality is prevalent among 19.2% [5]. Poor sleep (as defined by short duration or poor quality sleep) [6] has been associated with adverse emotional (eg, stress and depression), cognitive (eg, memory and cognitive performance), and physiological (eg, pain) symptoms in previous research [7,8]. Poor sleep has also been associated with obesity [9], diabetes [10], hypertension [11], cardiovascular disease [8,11], reduced quality of life [12], depression [12], and mortality [13]. The broad-ranging consequences of poor sleep show the need for interventions to improve sleep quality and duration.

Exogenous melatonin supplements are a widely available and commonly used intervention to treat poor sleep [14]. Several clinical trials have been conducted demonstrating the efficacy of exogenous melatonin in treating sleep disorders regardless of the etiology of the disorder [15-18]. However, clinical trials of melatonin for sleep have found significant heterogeneity in the effectiveness of melatonin interventions [14,19,20]. This is supported by a systematic review that assessed melatonin’s effect on several dimensions of sleep across 23 clinical trials and identified significant between-study heterogeneity [20]. These findings suggest that not all participants benefit equally from treatment with melatonin.

This heterogeneity in treatment response for individuals using exogenous melatonin suggests that participants who wish to improve their sleep may benefit from personalized N-of-1 trials [21,22]. Personalized N-of-1 trials are a single-case research approach designed to help patients select the treatment that works best for them as an individual. A personalized N-of-1 trial consists of multiple crossover intervention periods alternating between treatment(s) and control with continuous data collection on outcomes of interest [23,24]. Using single-case statistical methods, participants in personalized N-of-1 trials can evaluate which treatment (or treatments) works best for them on the outcome (or outcomes) that are most important to them. Personalized trials are designed to provide patients with high-integrity, evidence-based information uniquely relevant to the outcomes and values important to them so they may identify efficacious treatments [25]. Furthermore, personalized N-of-1 trials present a significant opportunity to determine the heterogeneity of treatment effects (HTEs) for a particular treatment. Although heterogeneity in treatment response has often been found for treatments (including melatonin), researchers are seldom able to calculate formal HTEs by comparing within and between participant treatment effects [26]. A series of personalized N-of-1 trials presents an ideal opportunity to formally measure the HTE of particular doses of melatonin [26,27]. High levels of HTE for doses of melatonin will indicate that future personalized N-of-1 trials of melatonin may be useful in a clinical context, while low levels of HTE suggest that the magnitude of treatment response does not vary greatly among participants.

Preliminary interviews suggest that personalized trials to improve poor sleep are a high priority among both patients and clinicians [23,28,29]. Despite the potential benefit of personalized N-of-1 trials, they are not common in clinical practice [23,28-30] due to the effort, participant burden, and cost required for implementation [31]. In order for personalized trials of melatonin for sleep to be more widely used, researchers need to identify a way to administer personalized N-of-1 trials in a manner that is feasible and acceptable to study participants. Some potential methods include the use of digital recruitment, remote intervention delivery, and online survey assessment. These methods aligned with increased emphasis on personalized medicine approaches for the improvement of sleep health [32,33].

This study evaluates the feasibility, acceptability, and effectiveness of a series of personalized N-of-1 trials of 2 doses of melatonin (3.0 mg and 0.5 mg, respectively) and a placebo supplement among 60 participants with self-reported poor sleep. Using commercially available supplements, medication adherence devices, an online survey assessment, and wearable technologies, we examined the overall effect of 2 doses of melatonin relative to usual care (ie, placebo) for each participant and aggregated among all participants. Furthermore, using statistical methods and implementation procedures developed in previous personalized N-of-1 trials [27], we identified the HTE for 3.0 mg and 0.5 mg of melatonin. Results from this study will determine whether digital delivery of these interventions is feasible and acceptable for participants with self-reported poor sleep and will allow clinicians to identify whether digital delivery of melatonin can effectively allow patients to manage their sleep.

## Methods

### Study Design

This study consisted of 60 digital, personalized N-of-1 crossover trials comparing the effects of 3.0 mg and 0.5 mg of melatonin versus placebo for poor sleep. The trial was comprised of a 2-week baseline assessment period and a 12-week intervention period. Before beginning the study, participants were provided with the necessary supplements, 3 Nomi by SMRxT smart pill bottles to contain the supplements, and a Fitbit Charge 5 (Google) activity tracker. The Nomi smart pill bottle is a medication adherence device that uses a weight sensor and cellular signals to measure and transmit medication adherence data in real-time.

During the 2-week baseline assessment period, participants did not take any melatonin or placebo pills and were asked to manage their sleep as they usually would. Participants were also encouraged to wear their Fitbit activity tracker and complete ecological momentary assessment (EMA) measures of fatigue, pain, concentration, confidence, mood, and stress delivered by text message 3 times daily, at randomized times. Each morning, participants completed an additional text-message survey assessing their sleep quality from the previous night.

Following baseline, participants were randomized into 1 of 2 treatment orders comprised of six 2-week intervention blocks of 3.0 mg melatonin, 0.5 mg melatonin, or placebo pills. Following the intervention, each participant was provided with a report summarizing the results of their personalized N-of-1 trial. At the end of the study, participants completed a survey containing satisfaction measures and the System Usability Scale (SUS). An example of the participant timeline can be found in Figure S1 in Multimedia Appendix 1. Study recruitment began in April 2022 and was completed in May 2023. Additional details about the study design can be found in the trial protocol [34].

### Study Population and Recruitment

This was a digital, remote clinical trial. Participants were recruited digitally from several states within the United States. Recruitment methods included internet and social media posting such as Facebook (Meta) and Google advertisements. In addition, online posts to communities on platforms such as Reddit, Craigslist, and LinkedIn were used to appeal to individuals with poor sleep quality. With 93% of the US adults reporting using the internet, 69% reporting ever using Facebook, and 18% reporting using Reddit, usage of online recruitment methods significantly broadened the potential span of our outreach [35]. Emails and advertisements were also distributed through the Northwell Health system, consisting of approximately 85,000 employees [36].

All potential participants identified as having poor sleep for at least the past month using the Insomnia Symptom Questionnaire [37]. Poor sleep for this study was defined by self-reported difficulty falling asleep, staying asleep, and/or feeling refreshed by sleep 3 to 7 times per week, with 1 or more of those symptoms lasting for a period of at least 4 weeks. Participants were also required to report that 1 or more poor sleep quality symptoms bother them “quite a bit” or “always” to meet eligibility criteria.

### Ethical Considerations

#### Ethical Review

This study protocol was approved by the ethics committee and Northwell Health Institutional Review Board (protocol 21-0239). Only IRB-approved team members were allowed access to identifiable data. Participant data were stored in IRB-approved secure databases.

#### Consent

Study recruitment materials directed participants to an information screen with details about the study and digital screening measures containing questions regarding study inclusion and exclusion criteria. If study inclusion criteria were met, eligible individuals were provided with a link to an electronic consent form and a short video explaining details of the study protocol and consent form. Consent for study participation was obtained electronically. Participants who had questions regarding the study details or consent process were offered a 30-minute phone call with study staff. All participants were aware the study intervention used 2 doses of melatonin and a placebo. Contact information for the study team was provided to potential participants throughout the consent process.

#### Compensation

After completing all components of the study (ie, successful completion of a research team-approved intervention period, submission of a satisfaction survey, and returning the Nomi devices), study participants were mailed a US $100 payment card (ClinCard). In addition, as a thanks to study participants for their participation, they were allowed to keep their Fitbit Charge 5 (a value of US $150).

### Interventions

Participants each received 3 Nomi pill bottles labeled A, B, and C. Bottles labeled with “A” contained 28 pills of 3.0 mg melatonin, bottles with “B” contained 28 pills of 0.5 mg melatonin, and bottles with “C” contained 28 pills of a placebo. All melatonin and placebo doses were provided for this study by Pure Encapsulations LLC, a National Sanitation Foundation– and Good Manufacturing Practices–registered laboratory with a long history of contributions to clinical research [38-40]. The placebo was 100% cellulose powder contained in a capsule, and the capsules of each placebo or supplement were color-matched and designed to be identical. Participants were blind to which capsule was stored in each bottle. The authors selected 3.0 mg and 0.5 mg doses to compare higher and lower doses of melatonin. Previous research has identified 0.3 to 1.0 mg as a low dose of melatonin capable of improving sleep [41]. Doses ranging between 2.0 and 6.0 mg of melatonin have also been found to be effective for improving sleep [42,43]. The doses of 3.0 mg, 0.5 mg, and placebo were selected in this study as we were able to obtain identical capsules for all 3 pills.

Participants were assigned 1 of 2 treatment orders: ABCCBA and CBAABC. Participants received an intervention schedule indicating which bottle labeled “A,” “B,” or “C” they should use for which weeks in the trial. During the intervention, participants received nightly reminder text messages instructing them to take a pill from the appropriate bottle (eg, “Take a pill from Bottle A”). All participants were discouraged from taking other melatonin products while enrolled in the trial. In total, 30 participants were assigned to each treatment order with study randomization conducted by the statistical team using Python to help randomize a list of each treatment order of 30. Randomization allocation was concealed from the study coordinators and investigators, and treatment orders were assigned by the statistical team only after minimum protocol adherence thresholds were achieved by each individual participant during baseline. The participant timeline in Figure S1 in Multimedia Appendix 1 shows these 2 treatment orders. During all study periods, participants were encouraged to wear the Fitbit device 24 hours a day and answer survey measures.

### Adherence

During the baseline period, the study team assessed participant adherence to the protocol. Adherence was evaluated using the Fitbit tracker and survey completion. Participants who wore the Fitbit for 12 or more hours per day and for at least 180 minutes while sleeping were defined as adherent to Fitbit wear [44,45]. Submission of a survey before the end of the 2-week baseline period was defined as adherent to survey completion. Participants who did not demonstrate adherence to 80% of study measures during baseline did not progress to the intervention.

### Participant Report

Participants who completed the trial received a 15-page personalized report summarizing their trial results by showing the impact of 3.0 mg melatonin, 0.5 mg melatonin, and placebo on their Fitbit-recorded sleep duration. The participant report also presented summaries and results for other sleep- and wellness-related outcomes including fatigue, stress, pain, and physical activity that may help the participant identify whether melatonin is useful to help their sleep or other well-being goals. Participants were also provided with summaries of their adherence to study measures and to the intervention. All reports were generated automatically but were reviewed by multiple study team members before being sent to participants. A similar version of the participant report in a series of personalized trials for chronic lower back pain is described and displayed in a study by D’Angelo et al [46].

### Primary Outcome

The primary outcome of this study was feasibility measured using the 10-item SUS [47] and acceptability as measured by an 18-item satisfaction survey. The SUS is a validated questionnaire that assesses the usability of a system with 10 items on a 5-point Likert scale rated from “strongly disagree” (1) to “strongly agree” (5) [5,48,49]. To obtain a total score, item scores are multiplied by 2.5 and summed to generate a total score ranging from 0 to 100, with higher scores indicating a greater level of usability. The SUS has been validated in multiple contexts [48,49] and has also been used for previous series of personalized N-of-1 trials [45,50]. As participant usability of a personalized trial is essential for a personalized N-of-1 design to be successful, the SUS is used as a feasibility measure. Analyses of the SUS include mean total scores and individual item scores with SDs. Mean SUS scores of ≥68 have been identified as “acceptable” levels of usability while scores ≥80 have been identified as “excellent” levels of usability [51]. We compared participant scores from this study to these established standards as well as SUS scores for other comparable trials to identify the feasibility of this series of personalized trials.

Participant satisfaction with the trial was measured using a 6-item survey rated on a scale of 1 (“not very satisfied”) to 5 (“very satisfied”). This survey measures participant satisfaction with specific elements of the trial (eg, the Fitbit activity tracker, SMS text messaging for reminders and surveys, and the personalized trial design). Participants also received a 7-item survey assessing their experience with the trial overall (eg, trial onboarding and informational videos) rated on a scale of 1 (“strongly disagree”) to 5 (“strongly agree”). Means and SDs for each item were represented, and frequencies of each participant response were depicted graphically. All satisfaction survey measures were structured similarly to previous series of personalized N-of-1 trials [44,45] but had the specific text altered to fit this trial design.

### Secondary Outcomes

The secondary outcomes included sleep duration measured using the Fitbit wearable activity tracker and sleep quality measured using a modified version of the consensus sleep diary (CSD). Nightly sleep duration was calculated using Fitbit’s proprietary algorithm with participant information from the Fitbit Charge 5 device. Participant Fitbit activity tracker data were retrieved using the online portals N1thrive and Fitabase. These portals allow for extraction of minute-to-minute activity data as well as daily summaries of outcomes including sleep duration and steps count. Although previous meta-analyses indicate that Fitbit activity tracking devices may overestimate sleep duration [52], the personalized N-of-1 design allows for within-participant comparisons of sleep durations between intervention periods. The CSD is a validated survey instrument used to assess and track intervention effects on sleep symptoms, quality, and satisfaction [53]. This study used a modified version of the CSD to assess perceived sleep quality using a 5-point Likert scale rated from “very poor” to “very good,” with higher scores indicating better sleep. The CSD was delivered daily via text message each morning within an hour of the participant’s self-reported typical wake time. Means and SDs of participant sleep duration and sleep quality were reported for baseline and each of the intervention periods (3.0 mg melatonin, 0.5 mg melatonin, and placebo). Paired samples *t* tests were used to compare mean participant sleep duration and sleep quality during the intervention periods to baseline. For analyses of self-reported sleep quality, participant responses were change log-transformed to comply with the assumptions for a normal distribution in a paired samples *t* test.

Additional secondary outcomes measured included self-reported EMA fatigue ratings and self-reported EMA stress ratings. EMA fatigue and stress were assessed using single-item measures adapted from the numeric pain rating scale [54]. These EMA measures were administered 3 times daily via SMS text message asking participants to rate their fatigue and stress in the current moment on a scale of 0-10. The EMA assessments were delivered randomly throughout the participant’s self-reported sleep and wake times. Paired samples *t* tests were used to compare ratings of EMA fatigue and EMA stress between baseline and each of the intervention periods (3.0 mg melatonin, 0.5 mg melatonin, and placebo).

The effect of each intervention (3.0 mg melatonin, 0.5 mg melatonin, and placebo) on sleep duration, EMA fatigue, and EMA stress was examined using generalized linear mixed models with an autoregressive model, which was used to account for possible linear trends and autocorrelation between outcome ratings over time. These models provided estimates of the effects of each treatment on each secondary outcome across all participants. We conducted additional analyses at the individual participant level to identify the effects of each intervention for each participant. These regressions also used generalized least squares analyses with autoregressive models. Using these regressions, we were able to generate estimates of the effect of each intervention on each outcome for every participant individually. Due to low levels of variation in self-reported sleep quality, participant ratings were averaged over each week, and a linear mixed model was conducted using these weekly averages for the overall sample and for each individual participant.

### HTEs

To support the utility of conducting a series of N-of-1 trials of melatonin for poor sleep, we examined the HTE for both 3.0 mg and 0.5 mg of melatonin. The HTE for both doses of melatonin was examined for 4 outcomes (ie, sleep duration, sleep quality, EMA fatigue, and EMA stress) using methodology previously developed for analyses of N-of-1 trials [27]. Briefly, we modeled the effects of 3.0 mg and 0.5 mg of melatonin relative to placebo on outcomes using linear mixed models with first-order autocorrelation autoregressive model. We then compared 2 models of the effects for 3.0 mg and 0.5 mg of melatonin versus placebo (1 with a random intercept only and 1 with random slopes) using a likelihood ratio test. The random slope model accounts for HTE, whereas the random intercept model does not. The results of this likelihood ratio test were then used to determine whether HTE exists for a particular treatment on a particular outcome. We also calculated an index of heterogeneity to quantify the HTE for each treatment on each outcome [27,55]. This index is used to interpret the utility of N-of-1 trials; higher values indicate that an N-of-1 trial may be more useful for examining the effects of a particular treatment on a particular outcome.

### Sample Size Calculation

The sample size of 60 participants was chosen to ensure a sufficient number to obtain a preliminary assessment of the feasibility of this series of personalized trials of melatonin therapy for sleep. With 60 participants receiving the intervention and use of a single sample binomial test with α set to 2.5% significance, we will have approximately 90% power if 70% (42/60) of participants complete the trial. With 57 participants completing the outcome, we have greater than 90% power.

## Results

### Enrollment and Sample Characteristics

**Figure 1.**
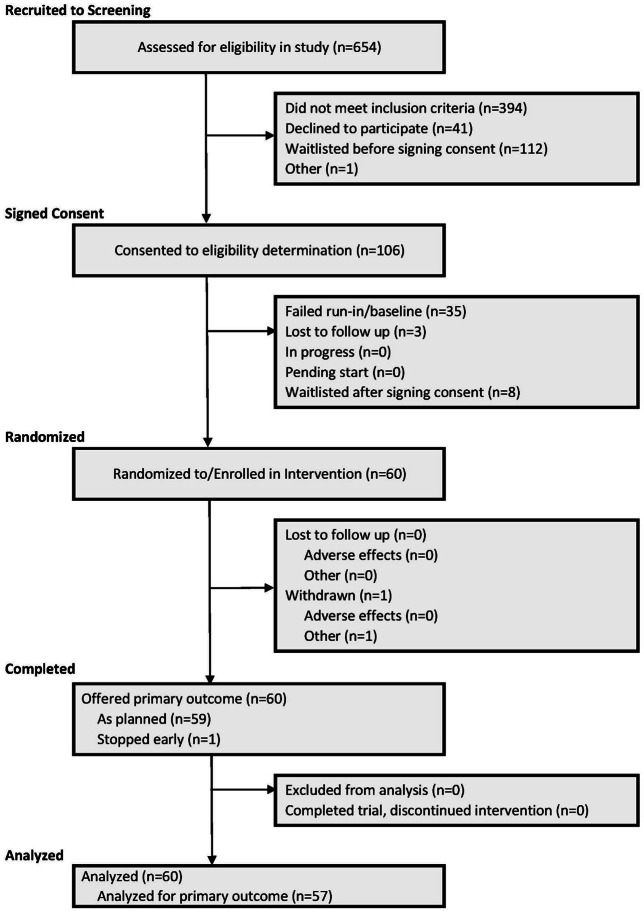
CONSORT (Consolidated Standards of Reporting Trials) extension for reporting N-of-1 (CENT) flow diagram.

In total, 654 participants were screened for the study, yielding an enrolled sample of 60 participants. Characteristics of the enrolled sample can be found in Table 1. Full details regarding study screening and enrollment can be found in the CONSORT (Consolidated Standards of Reporting Trials) diagram (Figure 1). Of this sample, 57 participants completed the primary outcome of the SUS. In this sample of 57 individuals, the mean age was 41.6 (SD 12.8) years, sex was 53% (n=30) female, 58% (n=33) White, and 18% (n=10) were Hispanic. Participant characteristics did not differ between the 2 treatment orders. Full characteristics of this sample can be found in Table S1 in Multimedia Appendix 1.

**Table 1.** Baseline characteristics of the sample (N=60).

Variable	Total sample	Treatment order 1 (n=30)^a^	Treatment order 2 (n=30)^b^	*P* value^c^
Age (y), mean (SD)	41.1 (12.7)	42.9 (13.1)	39.4 (12.3)	.29
Sex, n (%)				.30
Female	32 (53)	18 (60)	14 (47)	
Male	27 (45)	11 (37)	16 (53)	
Other	1 (2)	1 (3)	0 (0)	
Race, n (%)				.97
Asian	7 (12)	4 (13)	3 (10)	
Black	7 (12)	4 (13)	3 (10)	
Mixed	3 (5)	2 (7)	1 (3)	
Other	7 (12)	3 (10)	4 (13)	
White	34 (57)	16 (53)	18 (60)	
Unknown	2 (3)	1 (3)	1 (3)	
Ethnicity, n (%)				.75
Hispanic	12 (20)	7 (23)	5 (17)	
Non-Hispanic	47 (78)	23 (77)	24 (80)	
Unknown	1 (2)	0 (0)	1 (3)	

a Treatment order 1: 3 mg melatonin, 0.5 mg melatonin, placebo, placebo, 0.5 mg melatonin, 3 mg melatonin.

b Treatment order 2: placebo, 0.5 mg melatonin, 3 mg melatonin, 3 mg melatonin, 0.5 mg melatonin, placebo.

c *P* values for comparisons of participant characteristics between treatment orders were obtained.

### SUS

**Figure 2.**
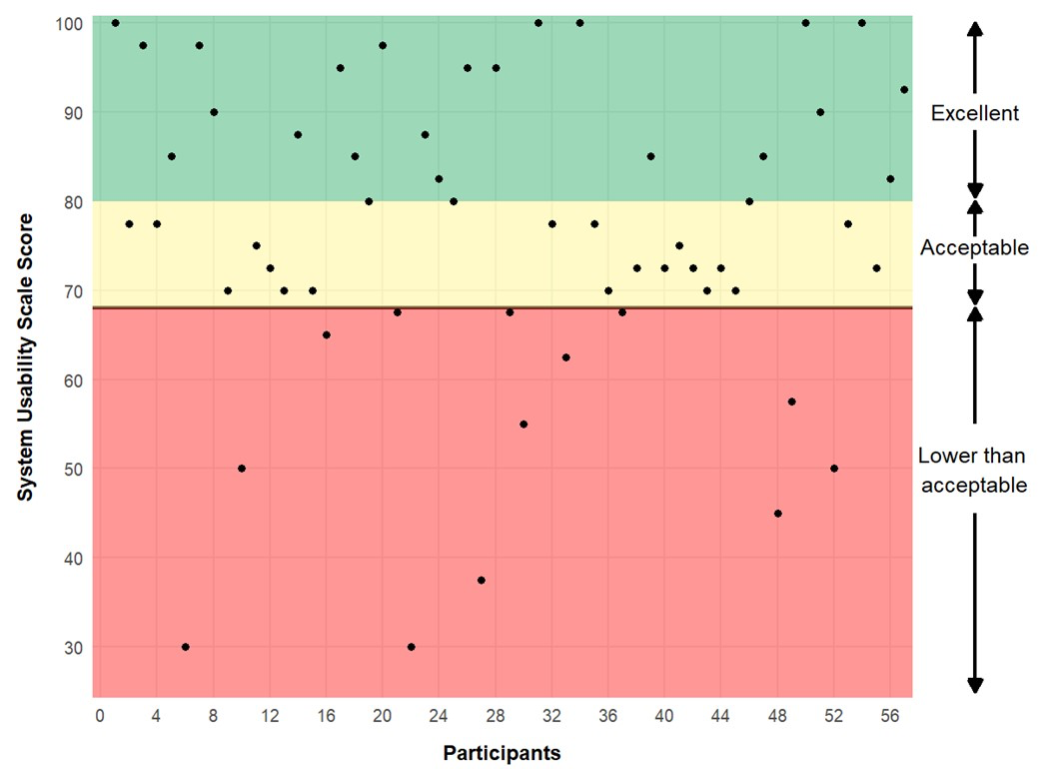
System Usability Scale (SUS) scores by participant (N=57*).* This figure displays each participant’s response to the SUS administered at the end of the trial. Ranges for excellent, acceptable, and less than acceptable ratings are based on the established literature for interpreting the SUS.

**Figure 3.**
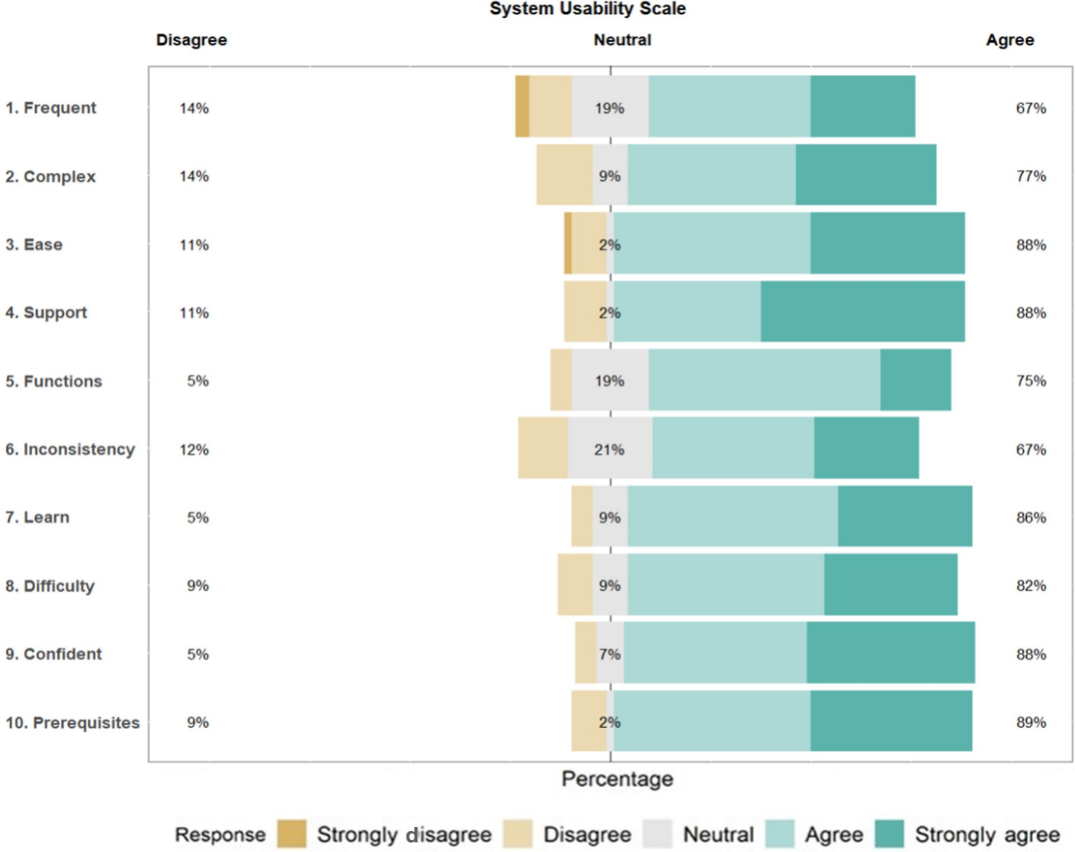
Distribution of responses for individual items on the System Usability Scale*.*

A total of 57 participants completed the primary outcome of the SUS. The mean SUS score across all participants was 76.27 (SD 17.09, Table 2), indicating that this trial had an acceptable level of usability (SUS total score≥70) [51]. Scores on the SUS ranged from 30 to 100 (Figure 2), indicating that several participants (13/57, 22.8%) found the usability and learnability of this series of personalized trials to be less than acceptable, while others (22/57, 38.6%) rated the usability of this series of personalized trials to be excellent. The most positively rated items from the SUS were “I do not think that I would need the support of a technical person to be able to use this system” (mean 3.28, SD 0.94) and “I felt very confident using the system” (mean 3.25, SD 0.81, Table 2). The least positively rated items were “I found the various functions in this system were well integrated” (mean 2.88, SD 0.76) and “I did not think there was too much inconsistency in this system” (mean 2.81, SD 0.97). Full distributions of responses for each item on the SUS can be found in Figure 3.

**Table 2.** System Usability Scale scores (N=57).

Measure	Values, mean (SD)	Range
System Usability Scale overall score	76.27 (17.09)	30-100
System Usability Scale individual items^a^		
I think that I would like to use this system frequently.	2.75 (1.07)	0-4
I did not find the system unnecessarily complex.^b^	1.02 (1.01)	0-3
I thought the system was easy to use.	3.14 (0.95)	0-4
I do not think that I would need the support of a technical person to be able to use this system.^b^	0.72 (0.94)	0-3
I found the various functions in this system were well integrated.	2.88 (0.76)	1-4
I did not think there was too much inconsistency in this system.	1.19 (0.97)	0-3
I would imagine that most people would learn to use this system very quickly.	3.14 (0.79)	1-4
I did not find the system very awkward to use.^b^	0.93 (0.88)	0-3
I felt very confident using the system.	3.25 (0.81)	1-4
I did not need to learn a lot of things before I could get going with this system.^b^	0.79 (0.86)	0-3

a Questions rated on a 5-point Likert scale from 0 “strongly disagree” to 4 to “strongly agree.”

b Items were initially reverse-coded but have been re-coded to be on the same scale as other items. The text of these questions has been revised from the original items to reduce confusion.

### Satisfaction

**Figure 4.**
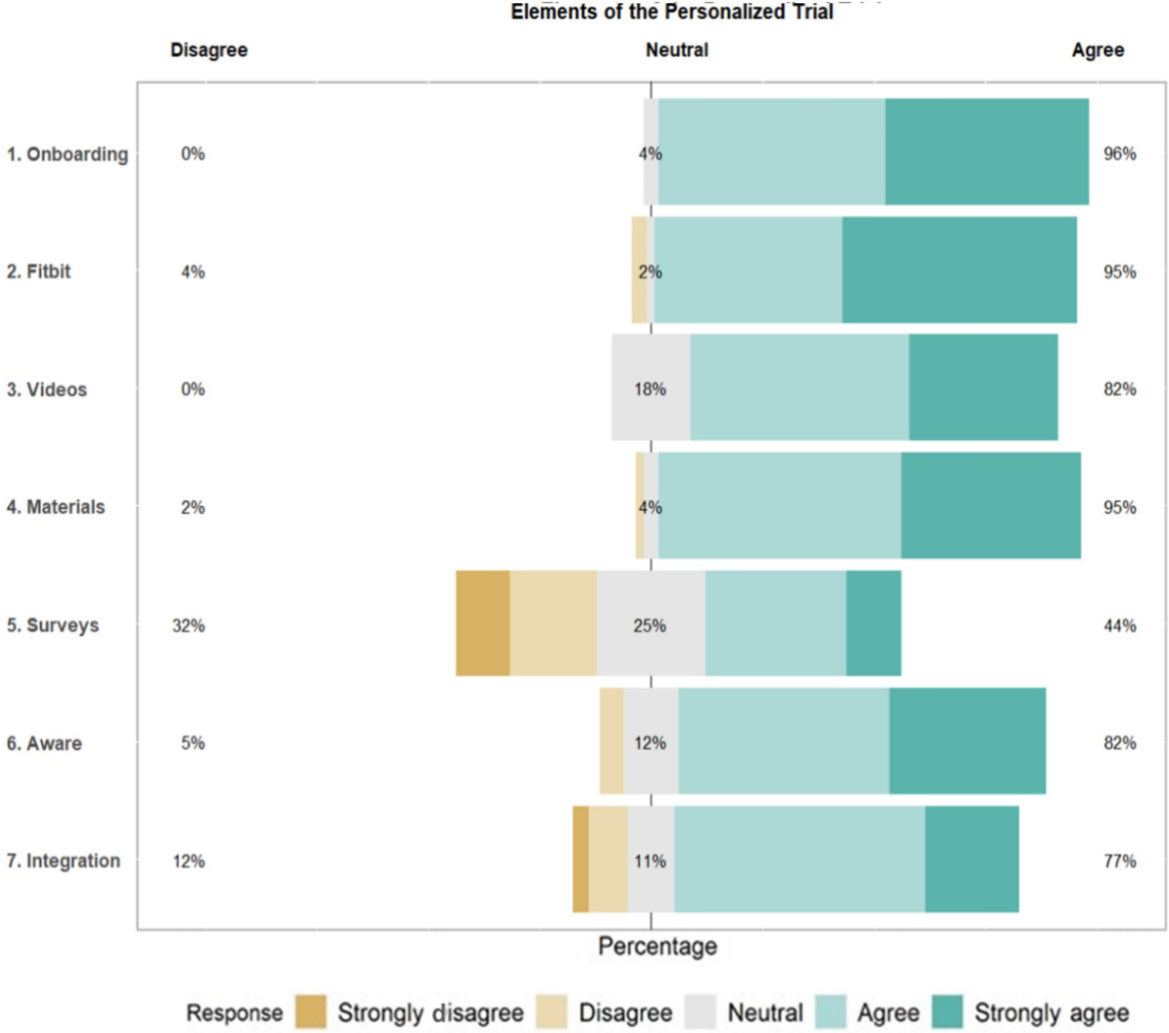
Participant satisfaction with elements of the personalized trial.

Participant satisfaction ratings of the trial were generally high on a 5-point scale (Figures4  5). The most positively rated items on the satisfaction survey were “use of the Fitbit to track your activity and sleep” (mean 4.58, SD 0.94) and “presentation of your results” (mean 4.51, 0.89). The least positively rated items on the satisfaction survey were “I enjoyed receiving daily text message prompts and surveys on my cell phone” (mean 3.12, SD 1.23) and “text messaging for survey questions” (mean 3.47, SD 1.48).

**Figure 5.**
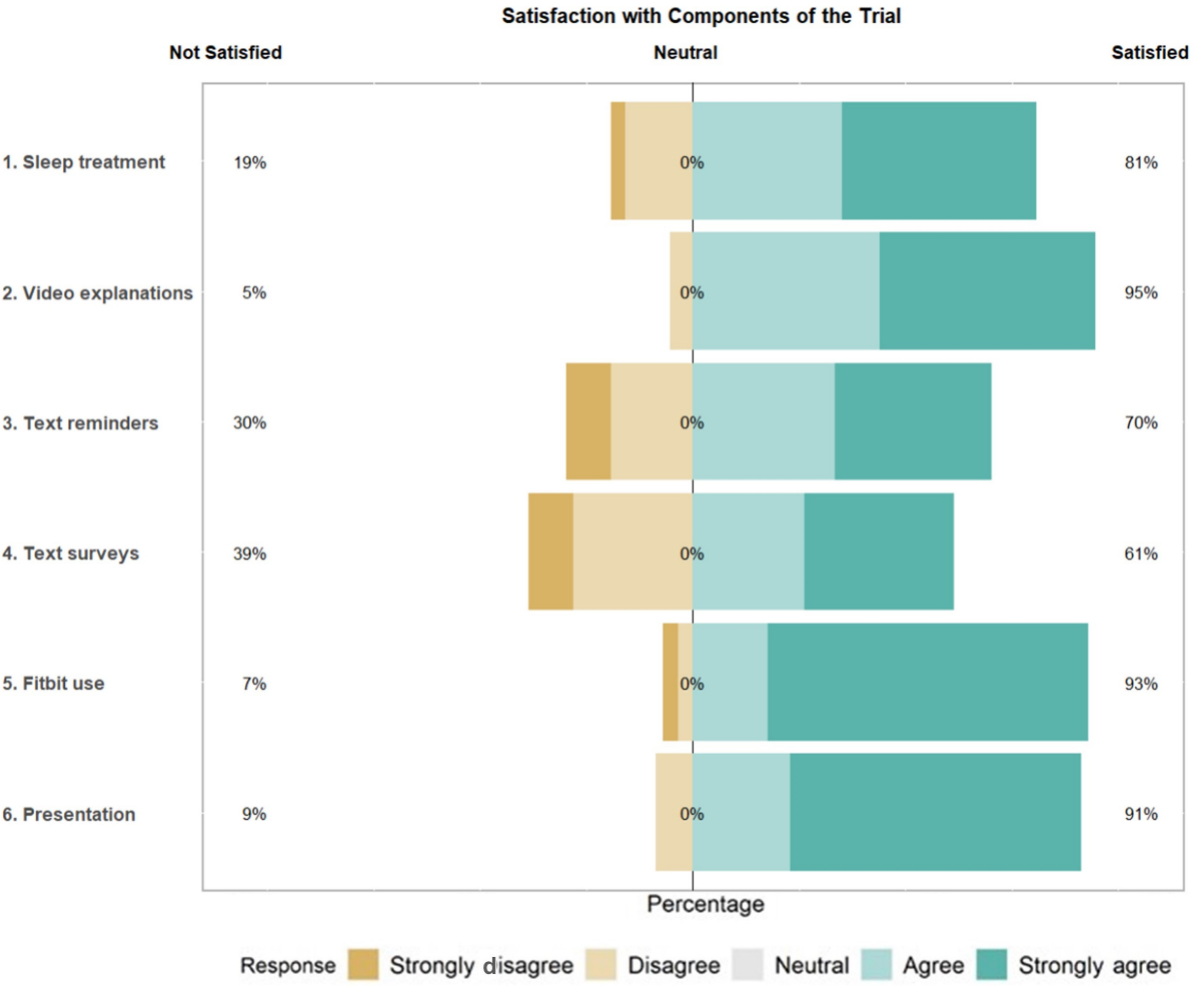
Participant satisfaction with components of the trial.

In addition, the majority of participants (36/57, 63%) stated they would “strongly recommend” the trial to others ( Multimedia Appendix 2). Only 3.5% (2/57) of participants stated they “would not recommend” the personalized trial to others. Participants mostly felt the trial was helpful; 12% (7/57) found the trial “extremely” helpful, 40% (23/57) rated the trial “very much” helpful, and 39% (22/57) found the trial “somewhat” helpful (Multimedia Appendix 3).

### Fitbit Sleep Duration

**Figure 6.**
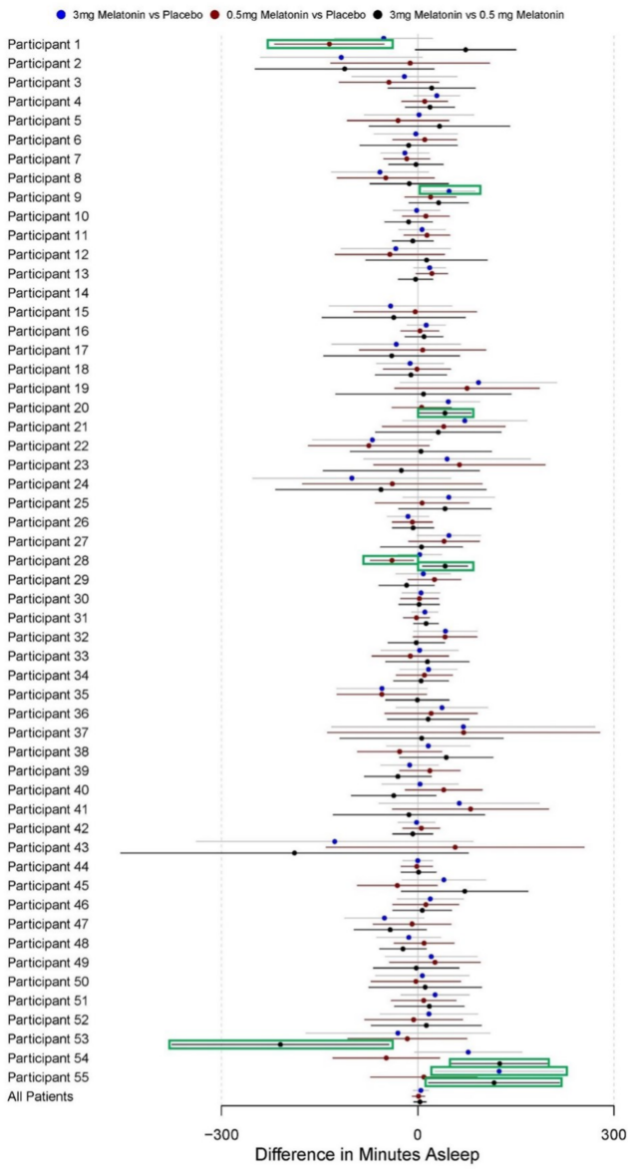
Difference in Fitbit-measured sleep duration by intervention period. These forest plots depict the results of autoregressive models of order 1 for each individual participant as well as pooled across all participants. CIs highlighted in green are for estimates that are statistically significant with *P*<.05.

At baseline, participants had 399 (SD 53) minutes of sleep per night, indicating participants started with roughly 6-and-a-half hours of sleep per night. Participant sleep durations were comparable during placebo (mean 398, SD 59 min), 0.5 mg melatonin (mean 398, SD 54 min), and 3.0 mg melatonin intervention periods (mean 402, SD 61 minutes) (Table 3). Paired samples *t* tests also showed no significant difference in mean sleep duration from baseline between placebo (*P*=.80), 0.5 mg melatonin (*P*=.90), and 3.0 mg melatonin (*P*=.70) intervention periods. Regression models in the pooled sample of participants showed no significant overall effects of 3.0 mg melatonin versus placebo (B=4.73, 95% CI −6.85 to 16.30), 0.5 mg melatonin versus placebo (B=1.23, 95% CI −8.28 to 10.75), and 3.0 mg melatonin versus 0.5 mg melatonin (B=3.40, 95% CI −6.24 to 13.03) (Table 4). Regression estimates for these treatment effects with 95% CIs can be found for overall sample and for each individual participant in Figure 6.

**Table 3.** Participant-reported outcomes by intervention period (N=55).

Outcome	Values, mean (SD)				Values, mean difference (95% CI)		
	Baseline	Placebo	0.5 mg melatonin	3 mg melatonin	Baseline versus placebo	0.5 mg versus baseline	3 mg versus baseline
Fitbit sleep duration (min/night)	399 (53)	398 (59)	398 (54)	402 (61)	−1.5 (−14.00 to 11.00)	−0.78 (−12.00 to 11.00)	2.6 (−9.60 to 15.00)
CSD^a^ sleep quality	3.13 (0.64)	3.38 (0.53)	3.44 (0.55)	3.35 (0.56)	0.25^b^ (0.11 to 0.39)	0.31^b^ (0.17 to 0.45)	0.21^c^ (0.07 to 0.35)
EMA^d^ fatigue	3.85 (1.95)	3.44 (2.00)	3.49 (2.07)	3.58 (1.98)	−0.41^c^ (−0.77 to −0.04)	−0.36^c^ (−0.72 to −0.01)	−0.29 (−0.61 to 0.03)
EMA stress	3.19 (1.86)	3.33 (1.96)	3.29 (1.86)	3.34 (1.78)	0.14 (−0.15 to 0.43)	0.10 (−0.14 to 0.33)	0.11 (−0.16 to 0.38)

a CSD: consensus sleep diary; measured on a scale of 1 to 5 with higher scores indicating better quality.

b *P* values for paired samples *t* tests: *P*<.001.

c *P* values for paired samples *t* tests: *P*<.05.

d EMA: ecological momentary assessment; measured on a scale of 1 to 10 with higher scores indicating higher levels (eg, a 10 would be the most stressed).

**Table 4.** Participant-reported outcomes by intervention period regression results.

Outcome	Values, B (95% CI)		
	3 mg melatonin versus placebo	0.5 mg melatonin versus placebo	3 mg melatonin versus 0.5 mg melatonin
Fitbit sleep duration (minutes/ night)	4.73 (−6.85 to 16.30)	1.23 (−8.28 to 10.75)	3.40 (−6.24 to 13.03)
CSD^a^ sleep quality	−0.04 (−0.13 to 0.06)	0.04 (−0.05 to 0.13)	−0.09 (−0.18 to −0.01)^b^
EMA^c^ fatigue	0.08 (−0.11 to 0.26)	0.04 (−0.12 to 0.20)	0.02 (−0.12 to 0.17)
EMA[Table-fn T4_FN3] stress	−0.07 (−0.25 to 0.11)	−0.04 (−0.17 to 0.10)	−0.04 (−0.21 to 0.14)

a CSD: consensus sleep diary; measured on a scale of 1 to 5 with higher scores indicating better quality.

b *P*<.05 in an autoregressive model

c EMA: ecological momentary assessment; measured on a scale of 1 to 10 with higher scores indicating higher levels.

### Sleep Quality

**Figure 7.**
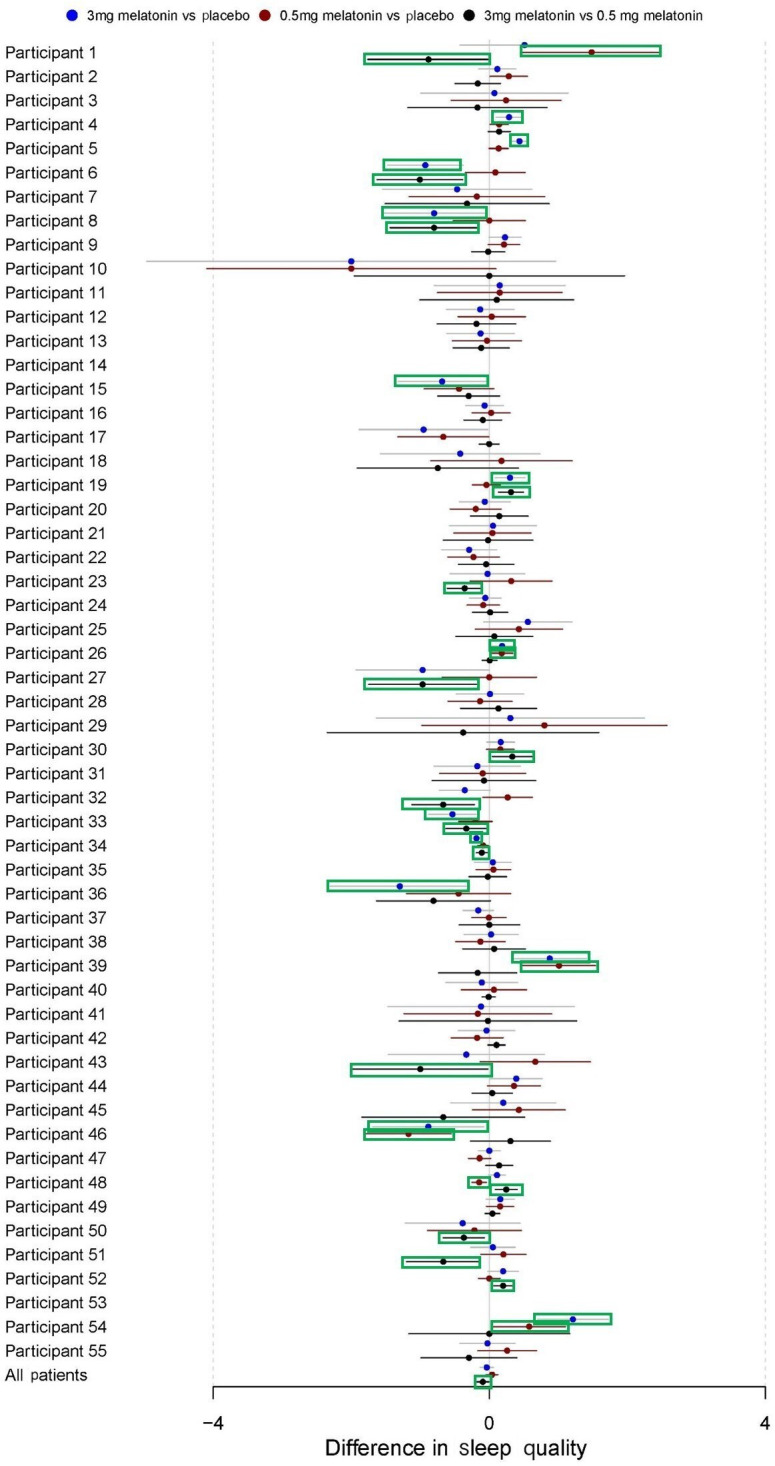
Difference in self-reported sleep quality by intervention period. These forest plots depict the results of autoregressive models of order 1 for each individual participant as well as pooled across all participants. CIs highlighted in green are for estimates that are statistically significant with *P*<.05.

During baseline, participants reported that their sleep quality was average (mean 3.13, SD 0.64 on a scale of 1 to 5, Table 3). Paired samples *t* tests also showed significant increases in mean sleep quality from baseline between placebo (*P*<.001), 0.5 mg melatonin (*P*<.001), and 3.0 mg melatonin (*P*=.005) intervention periods. However, regression models in the pooled sample of participants showed no significant overall effects for 3.0 mg melatonin versus placebo (B=−0.04, 95% CI −0.13 to 0.06) and 0.5 mg melatonin versus placebo (B=0.04, 95% CI −0.05 to 0.13). There was a small but statistically significant difference between 3.0 mg melatonin versus 0.5 mg melatonin (B=−0.09, 95% CI −0.18 to −0.01; Table 4). In Figure 7, regression estimates for these treatment effects with 95% CIs can be found for the overall sample and for each individual participant.

### EMA Measures of Fatigue and Stress

During baseline (mean 3.85, SD 1.95), participants also reported relatively low levels of fatigue (Table 3). Mean participant levels of fatigue decreased during placebo (mean 3.44, SD 2.00), 3.0 mg melatonin (mean 3.58, SD 1.98), and 0.5 mg melatonin periods (mean 3.49, SD 2.07). In addition, paired sample *t* tests showed small but statistically significant reductions in EMA fatigue between baseline and both 0.5 mg melatonin (mean difference −0.36, 95% CI −0.72 to −0.01; *P*=.045) and placebo periods (mean difference −0.41, 95% CI −0.77 to −0.04; *P*=.03). No significant differences were found between baseline and 3.0 mg melatonin periods in paired samples *t* tests (*P*=.08; Table 3). Regression estimates comparing EMA fatigue values between 3.0 mg melatonin (B=0.08, 95% CI −0.11 to 0.26) and 0.5 mg melatonin (B=0.04, 95% CI −0.12 to 0.20) versus placebo periods were also not statistically significant (Table 4).

**Figure 8.**
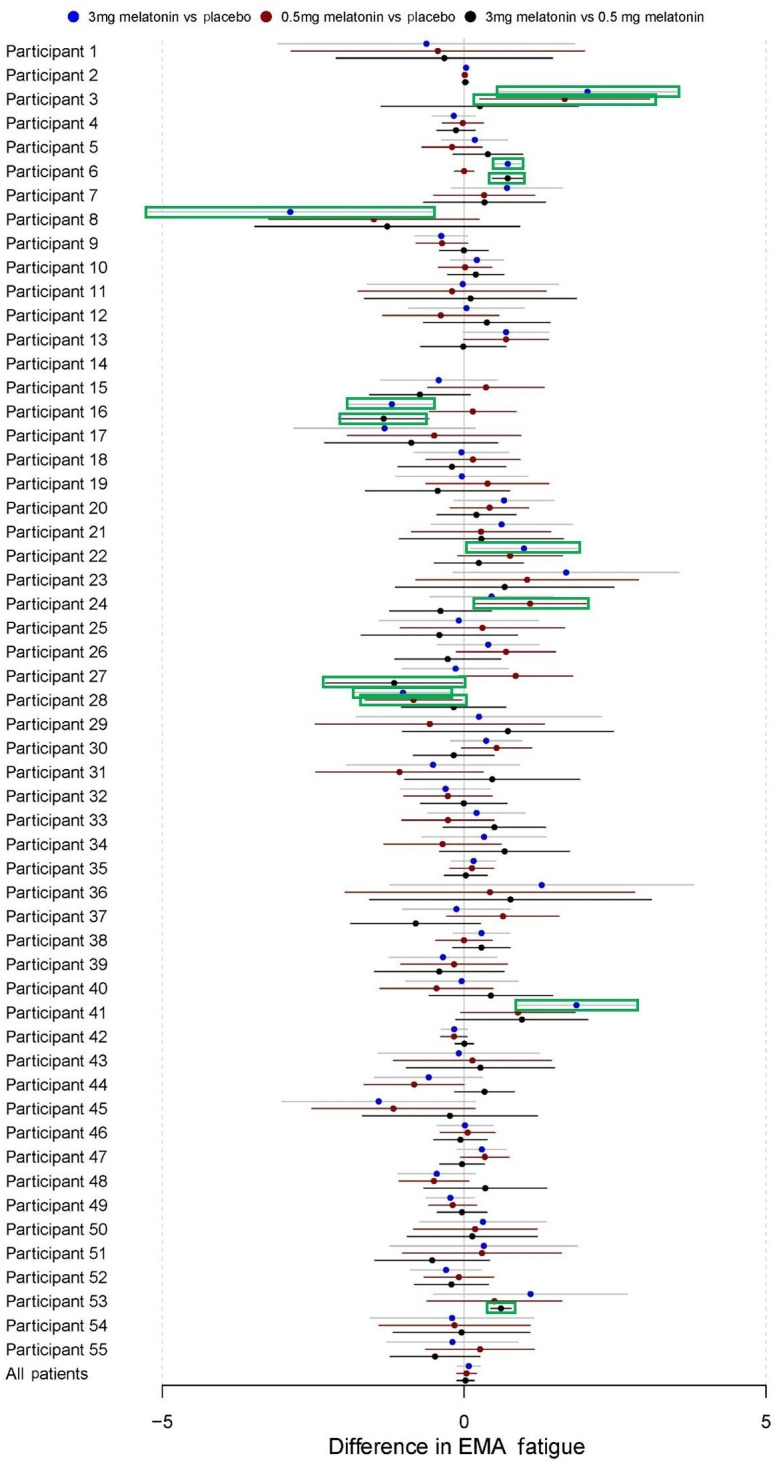
Difference in EMA fatigue. These forest plots depict the results of autoregressive models of order 1 for each individual participant as well as pooled across all participants. CIs highlighted in green are for estimated whichestimates that are statistically significant with *P*<.05. EMA: ecological momentary assessment.

Participants reported relatively low levels of stress during baseline (mean 3.19, SD 1.86), placebo (mean 3.33, SD 1.96), 0.5 mg melatonin (mean 3.29, SD 1.86), and 3.0 mg melatonin periods (mean 3.34, SD 1.78). Results from paired samples *t* tests did not show any significant difference between baseline and intervention periods (Table 3). Furthermore, there was no difference between treatment periods in the autoregression models conducted (Table 4). Figures8  9 present regression estimates for these fatigue and stress, respectively, with 95% CIs for the overall sample and for each individual participant.

**Figure 9.**
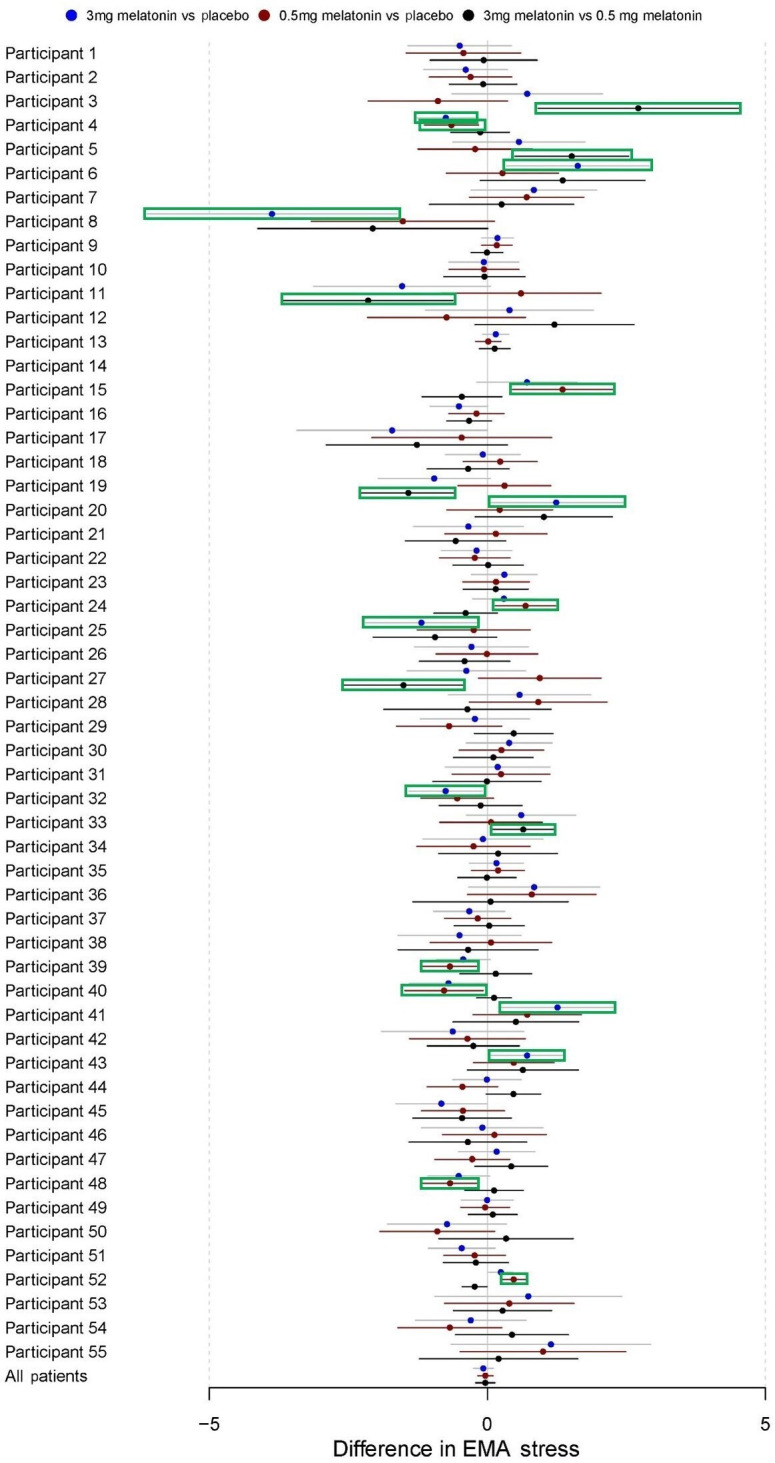
Difference in EMA stress. These forest plots depict the results of autoregressive models of order 1 for each individual participant as well as pooled across all participants. CIs highlighted in green are for estimated whichestimates that are statistically significant with *P*<.05. EMA: ecological momentary assessment.

### HTEs

Analyses of the HTE for placebo, 0.5 mg melatonin, and 3.0 mg melatonin showed that a random slope model was a better fit for the data than a random intercept for modeling the outcomes of sleep duration, EMA fatigue, and EMA stress (Table 5; likelihood ratio *P* value<.001). The SD of the treatment effect for 3.0 mg versus placebo and 0.5 mg melatonin versus placebo was also larger than the treatment effects. However, the indices of HTE for 3.0 mg versus placebo and 0.5 mg versus placebo were low for sleep duration, EMA fatigue, and EMA stress. These indices reflect the potential power for future personalized N-of-1 trials of each intervention for each outcome [27]. Based on these indices, future series of personalized N-of-1 trials will have statistical power ranging between 7% and 23% depending on the intervention and outcome (Table 5). This suggests that although a random slope model is more useful than a random intercept model for examining the effects of each dose of melatonin on these 3 outcomes, participants did not have a heterogeneous treatment response. Based on these preliminary findings, further N-of-1 trials of melatonin for sleep duration, EMA fatigue, and EMA stress are unlikely to be necessary in this population.

**Table 5.** Heterogeneity of treatment effect for secondary outcomes.

Outcomes	3 mg melatonin			0.5 mg melatonin			Overall		
	Treatment effect (3 mg melatonin versus placebo)	SD of treatment effect (3 mg melatonin)	HTE^a^ index for 3 mg melatonin	Treatment effect (0.5 mg melatonin versus placebo)	SD of treatment effect (0.5 mg melatonin)	HTE^a^ index for 0.5 mg melatonin	Between-subject SD of outcome	Within-subject SD of outcome	Likelihood ratio test *P* value^b^
Fitbit sleep duration (min/night)	4.52	25.85	0.23	0.96	10.60	0.07	53.04	110.25	<.001
EMA^c^ fatigue	0.08	0.45	0.17	0.05	0.33	0.10	1.97	1.42	<.001
EMA^c^ stress	−0.07	0.44	0.18	−0.03	0.20	0.07	1.92	1.23	<.001

a HTE: heterogeneity of treatment effect; this index ranges from 0.00 to 1.00 with higher scores indicating greater levels of heterogeneity.

b Results for this likelihood ratio test reflect an omnibus test comparison of a random intercept model of the treatment effect on the outcome versus a random slope model of the same treatment effect. A *P* value <.05 reflects a comparison in which the random slope model has a better fit.

c EMA: ecological momentary assessment; measured on a scale of 1 to 10 with higher scores indicating higher levels.

Analyses of the HTE for self-reported sleep quality were unable to be completed due to a lack of variance in participant responses. While a formal analysis of the HTE was not conducted for sleep quality, this lack of variance in participant responses also suggests that treatment effects of melatonin on sleep quality were not heterogeneous.

## Discussion

### Principal Findings

The results from this trial show that digital, remote personalized N-of-1 trials of melatonin for poor sleep are both feasible and acceptable to participants. However, the melatonin interventions were not significantly associated with changes in measures of poor sleep (including device-measured sleep duration and self-reported sleep quality). This suggests that, although this system of personalized N-of-1 trials can be successfully implemented among individuals with poor sleep, melatonin may not be an ideal intervention for a personalized N-of-1 trial design.

Participants rated the feasibility (defined using the SUS) as being acceptable. Although the mean SUS scores were lower than the previous series of personalized trials conducted by the authors [45,50], participant ratings were still higher than many other interventions with sleep as the primary outcome. In fact, usability ratings were comparable or better than previous interventions to improve sleep, including blue light therapy for sleep among patients with Parkinson disease (SUS median70, IQR 57.5-80.0) [56], a digital sleep diary intervention (SUS mean 74.6, SD 9.4) [57], a mobile health (mHealth) intervention to improve sleep among shift workers (SUS mean 62.7, SD 12.7) [58], an mHealth app to support the use of continuous positive airway pressure (CPAP) among patients with obstructive sleep apnea (SUS mean 79.4, SD 19.3) [59], an mHealth sleep monitoring device for management of sleep symptoms among older individuals (SUS mean 75.5, SD 10.2) [60], and a web-based decision aid for identifying treatment for obstructive sleep apnea (SUS mean 78.2, SD 15.1) [61]. Interestingly, Fitbit wearable activity trackers tend to have acceptable levels of usability on the SUS in previous research [62,63].

What is perhaps more interesting is the fact that both 3.0 mg and 0.5 mg doses of melatonin did not differ significantly from placebo in terms of Fitbit device-recorded sleep duration, self-reported sleep quality, and EMA measures of fatigue and stress. Furthermore, we did not find heterogeneity in the treatment response among participants. Notably, however, there were small but statistically significant differences in sleep quality and fatigue between the baseline and intervention periods. In addition, meta-analyses of the effect of melatonin on sleep duration have found an average increase of 12 minutes [14] and a significant improvement in sleep quality [20]. Both of these meta-analytic studies found heterogeneity in the treatment effects of melatonin [14,20]. Although this heterogeneity was identified in both meta-analyses using Cochran Q statistic (a method that has been called into question in recent literature) [64], our study found homogeneous, nonsignificant effects of melatonin versus placebo for many of our outcomes. This is especially surprising since the authors have previously found interventions used in series of N-of-1 trials to treat chronic lower back pain and fatigue had small overall effects but high levels of heterogeneity in treatment response for some interventions and outcomes [65,66].

While this finding is surprising, it is not unprecedented. Previous research has found that melatonin is most effective among individuals dealing with sleep restriction or altered sleep schedules (eg, individuals with jet lag and shift workers) [67]. This is logical given melatonin’s role in the regulation of circadian rhythm. However, the causes of poor sleep can be variable and complex. Lifestyle factors (eg, smoking, diet, and physical activity) [4,13], individual characteristics (eg, sex, BMI, socioeconomic status, and medical comorbidity) [4,13,68,69], and environment factors (eg, having children, marital status, irregular work hours, and noise) [4,70-72] can all influence sleep quality and duration. While melatonin may address how some of these factors impact circadian rhythm, melatonin supplements’ ability to address how environmental factors, such as noise, influence sleep will be difficult. However, we would hope that some participants in this trial would have poor sleep that was linked to a disruption of circadian rhythm. The lack of heterogeneity in our effects suggests that melatonin may not be a useful intervention for personalized N-of-1 trials that broadly recruit individuals with self-reported poor sleep.

### Strengths and Limitations

This study has several notable strengths. First, this series of personalized trials was implemented by a research team with extensive experience in the design and conduct of personalized N-of-1 research. The statistical team supporting the work of this trial has also developed methods for specifically analyzing personalized N-of-1 data and calculating HTE [27,73]. We believe this expertise has contributed to a well-designed and well-conducted series of personalized N-of-1 trials. Second, the use of digital, remote interventions, data collection, and dissemination of findings also means that the design of this study may be easily replicable. Third, feasibility and participant satisfaction with the trial were assessed using multiple metrics, including the SUS, a validated measure of usability used to evaluate other digital interventions. This measure was supplemented by satisfaction surveys that were tailored specifically to the design of this trial. These measures have allowed us to compare our series of personalized N-of-1 trials with other digital interventions for sleep while also collecting feedback specific to the design of this study.

Although this trial does have many strengths, there are also some limitations to consider. First, the outcomes used in this trial are all self-reported measures or measurements from a commercially available device. Both sleep duration [52] and sleep quality [74] can be measured using more accurate and precise measures to better capture dimensions of poor sleep. However, this trial uses readily available measures and devices designed to reduce participant burden. By using accessible and lower-cost tools to capture dimensions of poor sleep, the goal was to increase the feasibility of personalized N-of-1 trials for sleep. Regardless, more accurate measures of sleep duration and sleep quality may capture nuances of poor sleep that the measures in this trial do not. Second, participants entered the trial with generally high levels of self-reported sleep duration and sleep quality. The mean baseline sleep duration for the sample was 399 minutes. Although this duration is lower than the 7‐10 hours of sleep duration recommended for adults [75], sleep durations of ≤5 hours per night are mostly associated with adverse outcomes [76]. However, this average sleep duration falls within the definition of “short sleep” [76,77]. In addition, participants reported average levels of sleep quality at baseline (mean 3.13, SD 0.64). Individuals with poorer levels of sleep during baseline may have benefitted more from melatonin during the intervention period. Third, this trial only examined 2 doses of commercially available melatonin (3.0 mg and 0.5 mg). As discussed previously, doses of melatonin ranging from 0.3 mg to 6.0 mg have been used to successfully improve poor sleep [41-43]. The doses used in this trial may not have been optimal melatonin levels to influence poor sleep. Another limitation with commercially available melatonin is that some evidence suggests that the actual dose in melatonin supplements may vary significantly between lots [78]. We aimed to minimize this limitation by purchasing supplements from a single manufacturer in a single lot. A fourth and final limitation is that this trial was not designed to enroll a representative sample of individuals with poor sleep. Although there was a fairly diverse sample, to definitively say that the results from this series of personalized trials will generalize to all populations is impossible.

### Conclusion

The findings of this study show that personalized N-of-1 trials for poor sleep are highly feasible and acceptable. However, our results show that melatonin supplements did not significantly improve sleep duration or sleep quality. Furthermore, these treatment effects were not heterogeneous between participants. This suggests that, despite the increased prevalence of melatonin use in the United States [79], melatonin may not be broadly applicable as a treatment for poor sleep, especially in personalized N-of-1 trials.

## Supplementary material

10.2196/58192 Multimedia Appendix 1 Supplementary figures and tables, including the participant timeline and baseline characteristics of those who completed the System Usability Scale.

10.2196/58192 Multimedia Appendix 2 Descriptive statistics for satisfaction measures (N=57). All questions were assessed using a satisfaction survey at the end of the trial.

10.2196/58192 Multimedia Appendix 3 Participant ratings of the helpfulness of the trial (N=57).

10.2196/58192 Checklist 1 CONSORT extension for N-of-1 checklist.

## ABBREVIATIONS

CONSORT: Consolidated Standards of Reporting Trials
CSD: consensus sleep diary
EMA: ecological momentary assessment
HTE: heterogeneity of treatment effects
mHealth: mobile health
SUS: System Usability Scale
