# Supplementary material for: A Series of Personalized Melatonin Supplement Interventions for Poor Sleep: Feasibility Randomized Crossover Trial for Personalized N-of-1 Treatment
Source: JMIR Form Res. 2025 Sep 26;9:e58192. doi: 10.2196/58192 (PMC12468169; doi:10.2196/58192)
Supplement: Multimedia Appendix 1 [file formative-v9-e58192-s001.docx]

**Figure S1.** Participant timeline.


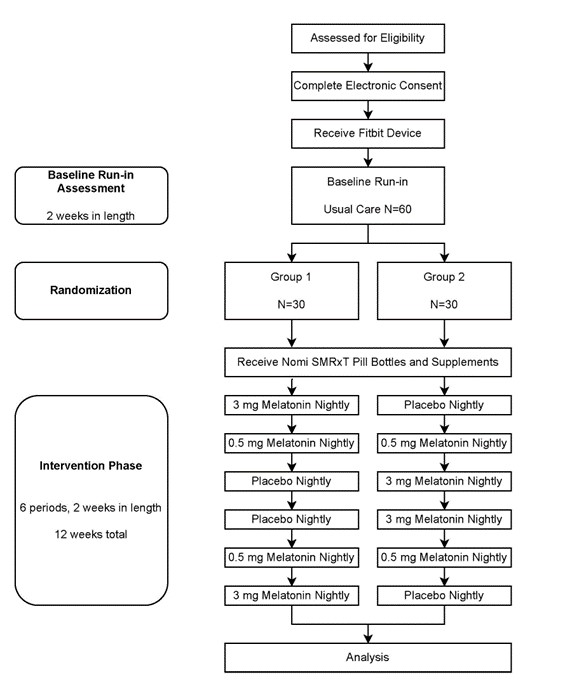


**Table S1.** Baseline characteristics of the sample who completed the primary outcome measure, the System Usability Scale (N=57).

| Variable | | Total sample | Treatment order 1 (n=29)^a^ | Treatment order 2 (n=28)^b^ | *P* value^c^ |
| --- | --- | --- | --- | --- | --- |
| Age (years), mean (SD) | | 41.6 (13) | 43.2 (13) | 40 (12) | 0.34 |
| **Gender, n (%)** | | | | | 0.14 |
|  | Female | 30 (53%) | 18 (62%) | 12 (43%) |  |
|  | Male | 26 (46%) | 10 (35%) | 16 (57%) |  |
|  | Other | 1 (2%) | 1 (4%) | 0 (0.0%) |  |
| **Race, n (%)** | | | | | >0.99 |
|  | Asian | 7 (12%) | 4 (14%) | 3 (11%) |  |
|  | Black | 7 (12%) | 4 (14%) | 3 (11%) |  |
|  | Mixed | 2 (4%) | 1 (4%) | 1 (4%) |  |
|  | Other | 6 (11%) | 3 (10%) | 3 (11%) |  |
|  | White | 33 (58%) | 16 (55%) | 17 (61%) |  |
|  | Unknown | 2 (4%) | 1 (4%) | 1 (4%) |  |
| **Ethnicity, n(%)** | | | | | 0.73 |
|  | Hispanic | 10 (18%) | 6 (21%) | 4 (14%) |  |
|  | Non-Hispanic | 46 (81%) | 23 (79%) | 23 (82%) |  |
|  | Unknown | 1 (2%) | 0 (0.0%) | 1 (4%) |  |

^a^Treatment order 1: 3mg melatonin, 0.5mg melatonin, placebo, placebo, 0.5mg melatonin, 3mg melatonin

^b^Treatment order 2: placebo, 0.5mg melatonin, 3mg melatonin, 3mg melatonin, 0.5mg melatonin, placebo

^c^P values for comparisons of participant characteristics between treatment orders were obtained from independent samples *t* tests for continuous variables and Fischer exact tests for categorical variables.
