# Supplementary material for: A Series of Personalized Melatonin Supplement Interventions for Poor Sleep: Feasibility Randomized Crossover Trial for Personalized N-of-1 Treatment
Source: JMIR Form Res. 2025 Sep 26;9:e58192. doi: 10.2196/58192 (PMC12468169; doi:10.2196/58192)
Supplement: Multimedia Appendix 2 [file formative-v9-e58192-s002.docx]

**Table S3.**

| Measure | Values, mean (SD) | Range |
| --- | --- | --- |
| Elements of the personalized trial |  |  |
| “I found the onboarding process (from the initial survey to getting my materials) for my personalized trial straightforward and easy to follow.” | 4.42 (0.57) | 3-5 |
| “I think my Fitbit charge 3 was easy to use.” | 4.44 (0.71) | 2-5 |
| “The informational videos helped me understand how to participate in this study.” | 4.16 (0.7) | 3-5 |
| “The materials I received in the mail were clear and easy to use.” | 4.33 (0.64) | 2-5 |
| “I enjoyed receiving daily text message prompts and surveys on my cell phone.” | 3.12 (1.23) | 1-5 |
| “I felt like I knew what was coming next in my personalized trial.” | 4.12 (0.83) | 2-5 |
| “My personalized trial was easy to integrate into my daily routine.” | 3.82 (0.98) | 1-5 |
| Satisfaction with components of the trialb |  |  |
| “Your personalized trial to improve sleep quality.” | 4.04 (1.19) | 1-5 |
| “Video explanations and demonstrations of study devices and procedures.” | 4.4 (0.75) | 2-5 |
| “Text messaging for reminders.” | 3.67 (1.42) | 1-5 |
| “Test messaging for survey questions.” | 3.47 (1.48) | 1-5 |
| “Use of the Fitbit Charge 3 to track your activity and sleep.” | 4.58 (0.94) | 1-5 |
| “Presentation of your results.” | 4.51 (0.89) | 2-5 |
