# Supplementary material for: A Series of Personalized Melatonin Supplement Interventions for Poor Sleep: Feasibility Randomized Crossover Trial for Personalized N-of-1 Treatment
Source: JMIR Form Res. 2025 Sep 26;9:e58192. doi: 10.2196/58192 (PMC12468169; doi:10.2196/58192)
Supplement: Multimedia Appendix 3 [file formative-v9-e58192-s003.docx]

**Table S4.**

| **Measure** | **Values, n (%)** |
| --- | --- |
| How much would you recommend this personalized trial to improve sleep quality to other persons? | |
| I would not recommend | 2 (4) |
| I would recommend a little bit | 19 (33) |
| I would strongly recommend | 36 (63) |
| Overall, how helpful was your participation in this study with respect to your sleep quality? | |
| Not at all helpful | 1 (2) |
| A little bit helpful | 4 (7) |
| Somewhat helpful | 22 (39) |
| Very much helpful | 23 (40) |
| Extremely helpful | 7 (12) |
