# Supplementary material for: A Series of Personalized Melatonin Supplement Interventions for Poor Sleep: Feasibility Randomized Crossover Trial for Personalized N-of-1 Treatment
Source: JMIR Form Res. 2025 Sep 26;9:e58192. doi: 10.2196/58192 (PMC12468169; doi:10.2196/58192)
Supplement: Checklist 1 [file formative-v9-e58192-s004.pdf]

CONSORT Extension for N-of-1 Checklist

Section/Topic	Number	CONSORT/ CENT Elements<br>Item	Page(s)
**Title and abstract**			
	1a	Identify as an "N-of-1 trial" in the title<br>***For series:*** Identify as "a series of N-of-1 trials" in the title	1
	1b	Structured summary of trial design, methods, results, and conclusions (for specific guidance see CONSORT for abstracts)	2
**Introduction**			
	2a.1	Scientific background and explanation of rationale	4
	2a.2	Rationale for using N-of-1 approach	4 to 5
	2b	Specific objectives or hypotheses	5
**Methods**			
	3a	Description of trial design (such as parallel, factorial) including allocation ratio	6
	3b	Important changes to methods after trial start (such as eligibility criteria), with reasons	N/A
	4a	Eligibility criteria for participants	7
	4b	Settings and locations where the data were collected	7
	4c	Whether the trial(s) represents a research study and if so, whether institutional ethics approval was obtained	7
	5	The interventions for each period with sufficient details to allow replication, including how and when they were actually administered	8 to 9
	6a.1	Completely defined pre-specified primary and secondary outcome measures, including how and when they were assessed	9 to 12
	6a.2	Description and measurement properties (validity and reliability) of outcome assessment tools	9 to 12
	6b	Any changes to trial outcomes after the trial commenced, with reasons	N/A
	7a	How sample size was determined	12
	7b	When applicable, explanation of any interim analyses and stopping guidelines	N/A
Randomisation:			
	8a	Whether the order of treatment periods was randomised, with rationale, and method used to generate allocation sequence	8 to 9
	8b	When applicable, type of randomisation; details of any restrictions (such as pairs, blocking)	8 to 9

	8c	Full, intended sequence of periods	8 to 9
	9	Mechanism used to implement the random allocation sequence (such as sequentially numbered containers), describing any steps taken to conceal the sequence until interventions were assigned	8 to 9
	10	Who generated the random allocation sequence, who enrolled participants, and who assigned participants to interventions	8
	11a	If done, who was blinded after assignment to interventions (for example, participants, care providers, those assessing outcomes) and how	9
	11b	If relevant, description of the similarity of interventions	8
	12a	Methods used to summarize data and compare interventions for primary and secondary outcomes	8 to 12
	12b	***For series:*** If done, methods of quantitative synthesis of individual trial data, including subgroup analyses, adjusted analyses, and how heterogeneity between participants was assessed, (for specific guidance on reporting syntheses of multiple trials, please consult the PRISMA Statement)	8 to 12
	12c	Statistical methods used to account for carryover effect, period effects, and intra-subject correlation	8 to 12
**Results**			
	13a.1	Number and sequence of periods completed, and any changes from original plan with reasons	13
	13a.2	***For series:*** The number of participants who were enrolled, assigned to interventions, and analysed for the primary outcome	13
	13c	***For series:*** losses or exclusions of participants after treatment assignment, with reasons, and period in which this occurred, if applicable	13
	14a	Dates defining the periods of recruitment and follow-up	Supplemental Figure 2
	14b	Whether any periods were stopped early and/or whether trial was stopped early, with reason(s).	N/A
	15	A table showing baseline demographic and clinical characteristics for each group	Table 1
	16	For each intervention, number of periods analysed.<br>***In addition for series:*** if quantitative synthesis was performed, number of trials for which data were synthesized	Supplemental Figure 2

	17a.1	For each primary and secondary outcome, results for each period; an accompanying figure displaying the trial data is recommended.	14 to 20
	17a.2	For each primary and secondary outcome, the estimated effect size and its precision (such as 95% confidence interval)<br>***In addition for series:*** if quantitative synthesis was performed, group estimates of effect and precision for each primary and secondary outcome	14 to 20
	17b	For binary outcomes, presentation of both absolute and relative effect sizes is recommended	14 to 20
	18	Results of any other analyses performed, including assessment of carryover effects, period effects, intra-subject correlation<br>***In addition for series:*** If done, results of subgroup or sensitivity analyses	14 to 20
	19	All harms or unintended effects for each intervention. *(for specific guidance see CONSORT for harms)*	N/A
**Discussion**			
Limitations	20	Trial limitations, addressing sources of potential bias, imprecision, and, if relevant, multiplicity of analyses	23 to 24
Generalisability	21	Generalisability (external validity, applicability) of the trial findings	23 to 24
Interpretation	22	Interpretation consistent with results, balancing benefits and harms, and considering other relevant evidence	21 to 23
**Other information**			
Registration	23	Registration number and name of trial registry	2
Protocol	24	Where the full trial protocol can be accessed, if available	25
Funding	25	Sources of funding and other support (such as supply of drugs), role of funders	25
